# Analysis of breast cancer subtypes by AP-ISA biclustering

**DOI:** 10.1186/s12859-017-1926-z

**Published:** 2017-11-14

**Authors:** Liying Yang, Yunyan Shen, Xiguo Yuan, Junying Zhang, Jianhua Wei

**Affiliations:** 1 School of Computer Science and Technology, Xidian University, Xi’an, Shaanxi 710071 China; 2 State Key Laboratory of Military Stomatology & National Clinical Research Center for Oral Diseases & Shaanxi Clinical Research Center for Oral Diseases, Department of Maxillofacial Surgery, School of Stomatology, The Fourth Military Medical University, Xi’an, Shaanxi 710032 China

**Keywords:** Breast cancer, Subtype, Classification, Biclustering, Gene expression profiles, Methylation

## Abstract

**Background:**

Gene expression profiling has led to the definition of breast cancer molecular subtypes: Basal-like, HER2-enriched, LuminalA, LuminalB and Normal-like. Different subtypes exhibit diverse responses to treatment. In the past years, several traditional clustering algorithms have been applied to analyze gene expression profiling. However, accurate identification of breast cancer subtypes, especially within highly variable LuminalA subtype, remains a challenge. Furthermore, the relationship between DNA methylation and expression level in different breast cancer subtypes is not clear.

**Results:**

In this study, a modified ISA biclustering algorithm, termed AP-ISA, was proposed to identify breast cancer subtypes. Comparing with ISA, AP-ISA provides the optimized strategy to select seeds and thresholds in the circumstance that prior knowledge is absent. Experimental results on 574 breast cancer samples were evaluated using clinical ER/PR information, PAM50 subtypes and the results of five peer to peer methods. One remarkable point in the experiment is that, AP-ISA divided the expression profiles of the luminal samples into four distinct classes. Enrichment analysis and methylation analysis showed obvious distinction among the four subgroups. Tumor variability within the Luminal subtype is observed in the experiments, which could contribute to the development of novel directed therapies.

**Conclusions:**

Aiming at breast cancer subtype classification, a novel biclustering algorithm AP-ISA is proposed in this paper. AP-ISA classifies breast cancer into seven subtypes and we argue that there are four subtypes in luminal samples. Comparison with other methods validates the effectiveness of AP-ISA. New genes that would be useful for targeted treatment of breast cancer were also obtained in this study.

**Electronic supplementary material:**

The online version of this article (doi:10.1186/s12859-017-1926-z) contains supplementary material, which is available to authorized users.

## Background

Breast cancer is a complex and heterogeneous disease and one of the leading causes of cancer-related death among women. The prognosis of breast cancer patients has been improved over time. However, further improvements in targeted treatment for breast cancer patients are expecting to solve the problem that why current therapy has effect only on a portion of the patients. A major milestone on the way to this goal is the definition of breast cancer molecular subtypes based on gene expression profiles: Basal-like [1], LuminalA, LuminalB, HER2-enriched and Normal-like [2–5], which are used in PAM50 [6]. SCMGENE and IntClust are also breast cancer classification system [7, 8]. SCMGENE includes only four subtypes which could not reflect the whole difference in expression profiles, while IntClust classifies the breast cancer into ten subclasses which needs further validation. Most studies performed gene expression analysis using a published ‘intrinsic gene list’ [6], which consisted of genes with significant variation in expression between different tumors, rather than between paired samples from the same tumor [4]. Recently, breast cancer are divided into subgroups according to expression patterns, especially LuminalA breast tumors [9].

Several approaches were used to analyze patterns in gene expression data [2, 10], such as hierarchical cluster which grouped samples based on the similarity of the expression across all genes. These traditional clustering approaches perform well only in finding global patterns. Many regulatory patterns, however, involve only a subset of genes and/or samples. For this reason, biclustering algorithms [11, 12] have been developed for biological data analysis to find local patterns in the data [13–15]. A bicluster is defined as a subgroup of genes that are co-expressed across only a subset of samples. Iterative signature algorithm (ISA) is a biclustering algorithm [16]. However, ISA biclustering results might be variable because seeds are selected randomly. Moreover, the samples’ number in every bicluster is similar since constant threshold is used, which can not reflect the ratio of each subtype in clinical diagnosis.

Epigenetic modification, such as DNA methylation, plays an important role in development, chromosomal stability and maintaining gene expression states [17]. In normal samples, the methylation status of CpG (Cytosine & Phosphoric acid & Guanine) sites were shown to unmethylated in CpG islands and methylated in gene body. It is proved that DNA methylation changes play a vital role in cancer initiation and progression [18, 19]. Especially, silencing of cancer suppressor genes was associated with promoter hypermethylation. Several recent studies show that breast cancer subtypes associate with methylation patterns [20]. Less is known about the relationship between DNA methylation and expression level in different breast cancer subtypes.

In this paper, a hybrid method, titled AP-ISA (Iterative Signature Algorithm based on Affinity Propagation), was proposed to classify breast cancer into subtypes, which integrated AP (Affinity Propagation) clustering [21, 22] and ISA (Iterative Signature Algorithm) [16]. AP-ISA embedded the result of AP clustering in ISA seed selection as prior knowledge. The aim of this study is to improve the classification performance of breast cancer subtypes and explore the association between DNA methylation level and gene expression in the subtypes. Experimental results validate the proposed method, which could contribute to targeted drug development and precision diagnosis.

## Methods

### Materials

The breast cancer dataset used in this study was derived from TCGA (The Cancer Genome Atlas) project [23], which consisted of 525 breast tumors and 22 normal breast samples. There are 17,815 genes in the dataset and we extracted 1906 genes using ‘intrinsic gene list’ [6]. DNA-methylation data was obtained from TCGA on the same samples. ER and PR information are also adopted to help the analysis. The datasets were stored at publicly available website (https://tcga-data.nci.nih.gov/docs/publications/brca_2012/) and intrinsic gene list can be obtained from publicly available website (http://ascopubs.org/doi/suppl/10.1200/jco.2008.18.1370).

### The design of the study

Biclustering is a method that finds sub-matrices inside a matrix on the basis of “local similarity” criterion. For gene expression data, sub-matrices are done simultaneously for genes and samples. Biclustering allows to obtain overlapping biclusters, in which a gene can be involved in different regulation patterns. Generally, ISA method is an iterative procedure using a random seed vector to start and its threshold are same for every seed. Among the existing biclustering algorithms [24], ISA performs effectively and efficiently. However, in ISA, initial seeds could influence biclustering results and the prior probability of subtype is not taken into account due to the lack of prior knowledge. When ISA is used to classify breast cancer, considering the existing problem, we put forward a modified ISA approach based on AP clustering, that is, AP-ISA. There are two important characteristics in AP-ISA. The first one is that, instead of random selection, seeds are produced based on the result of AP clustering, where the ratio of breast cancer subtypes in clinical diagnose could be adopted. Providing different thresholds for different seeds is the other characteristic of AP-ISA. We set smaller thresholds for the seed categories with bigger size, to guarantee that the biclusters with bigger size can be obtained, and vice versa. Therefore, the biclustering results could reflect the clinical diagnosing information.

### Iterative signature algorithm

Compared to other biclustering algorithms, ISA is effective to deal with gene expression data. It is a process to extract the TM (Transcription Module) [15, 16]. Each TM contains both a set of genes and a set of experimental conditions. The conditions of the TM induce a co-regulated expression of the genes belonging to this TM. It means, the expression profiles of the genes in the TM are the most similar to each other when compared over the conditions of the TM. Conversely, the patterns of gene expression obtained under the conditions of the TM are the most similar to each other when compared only over the genes of the TM. The degree of similarity is determined by a pair of threshold parameters. The ISA starts from a set of randomly selected genes or conditions, then iteratively refines the genes and conditions until they match the definition of a TM.

Considering a gene expression matrix E of size *m* × *n*, where *m* and *n* are the number of samples and genes, the ISA algorithm performs in the following way. Firstly, it creates a group of seeds, that is, a group of random sparse 0/1 vector of size *m*. For each seed, the following iteration is performed. We take a seed vector *c* ^0^ as example. The non-zero elements of *c* ^0^ are used to select a subset of the samples (rows of E). It also can use ‘smart seeding’, where the seeds are biased to start with certain sets of genes or samples based on prior knowledge. Row-normalized matrix *E*_*C*_ and column-normalized matrix *E*_*G*_ are calculated. *E*_*C*_ is multiplied by *c* ^0^, and the result is processed by threshold *t*_*G*_, to get the vector *g*_0_ with size *n*. The non-zero elements of *g*_0_ are used to select a subset of the genes (columns of E). In a similar way, *E*_*G*_ is multiplied by *g*_0_, and processed by threshold *t*_*C*_ in order to obtain the vector *c* ^1^ with size *m*. This procedure iteratively proceeds until either *g* ^(*i* − 1)^ and *g* ^(*i*)^, *c* ^(*i* − 1)^ and *c* ^(*i*)^are approximate enough according to convergence criteria, where *i* is the maximum of iteration times. The non-zero elements in *g* ^(*i*)^and *c* ^(*i*)^ are selected as genes and samples in the bicluster based on c^0^. If n seeds are initialized in the beginning, there will be n biclusters, from which some biclusters are selected according to the diversity as the final clustering results.

From the above procedure, it can be seen that there are two important parameters in ISA, which will affect the results. They are the two thresholds: *t*_*G*_ for columns that associates with genes and *t*_*C*_ for rows that is related to samples. For example, if the row threshold *t*_*C*_ is high, the biclusters will contain more similar samples. Lower threshold values, in turn, will provide bigger biclusters with less similar samples. In this work, we use R package isa2 to implement ISA algorithm [25].

### AP-ISA: Modified ISA based on AP clustering algorithm

Considering ISA algorithm is quite sensitive to the initial seeds, we innovatively use the result of AP algorithm as the prior knowledge for seed selection. Thus, AP-ISA, a modified ISA algorithm based on AP clustering, comes into being. AP is a clustering algorithm that takes similarity measures between pairs of data points as input. Real-valued messages are exchanged between data points until a high-quality set of exemplars and corresponding clusters gradually emerge [21]. Here the samples in AP clusters are used to select and classify useful seeds and further, to control the selection of thresholds, which guarantees that the biclusters’ size is reasonable compared with real distribution of breast cancer subtypes. The AP-ISA algorithm performs as follows.

- Step 1. AP clustering. For gene expression matrix E, AP takes a collection of real-valued similarities between samples as input. A parameter K is set. K is the desired number of clusters. AP clustering results are K sample subsets, which are denoted as *S*_*i*_ (*i* = 1, 2…K).
- Step 2. Seed selection and clustering. ISA algorithm is adopted to created 10,000 random sparse 0/1 vector of size m as seeds, where m is the number of samples. The seeds are gathered into K clusters to guarantee that, the seeds whose corresponding samples of non-zero elements are in the same AP cluster *S*_*i*_, are assigned to the same group *C*_*i*_. There are some seeds that violate the guarantee, which means that the corresponding samples of non-zero elements in the seeds are not in the same AP resulting cluster. Therefore, they cannot be allocated into any of the K resulting clusters. These seeds are deleted. We denote all remaining seeds as matrix *C* = *C*_1_ ∪ *C*_2_ ∪ ⋯ ∪ *C*_*K*_, where *C*_*i*_ (*i* = 1, 2, …., K) is the i-th seed group. Generally, the number of seeds in *C* is less than 10,000. For bigger scale cluster in AP results, bigger scale seed cluster will be obtained accordingly.
- Step 3. Biclustering. The seed matrix *C* and gene expression matrix *E* are used as input of the ISA process. The two thresholds *t*_*G*_ and *t*_*C*_ are set for each seed group respectively. For a seed *c* ^0^ (*c* ^0^ ∈ *C*), it multiplied by row-normalized matrix *E*_*c*_ and the result is processed by threshold *t*_*G*_ to get the vector *g* ^0^. In a similar way, column-normalized matrix *E*_*G*_ is multiplied by *g* ^0^, and processed by threshold *t*_*C*_. After this iterative procedure, a bicluster corresponding to *c* ^0^ is obtained. For each seed in *C*, one biclsuter will be produced. Finally, the biclusters with bigger diversity are chosen.

It is worth noting that the sample size of each bicluster *S*_*i*_ (*i* = 1, 2…K) represents the possibility of breast cancer subtypes happening in clinical diagnosis. The greater the number of samples in *S*_*i*_, the more seeds in *C*_*i*_ than in other seed groups (i = 1, 2….K). For bigger size of seed group, it is better to set smaller row threshold so that the biclusters will have more samples. Smaller size of seed group, in turn, should be matched with bigger row threshold for providing biclusters with less and more similar samples. The AP-ISA algorithm is described as follows.
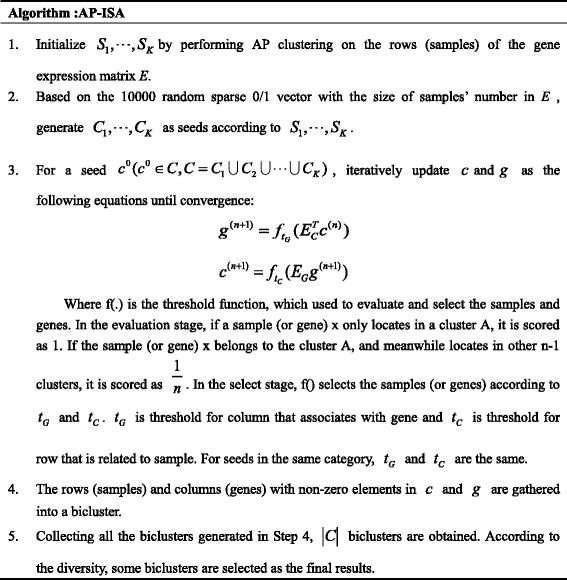


In brief, the main merits of AP-ISA are as follows. AP algorithm is adopted to capture the subtypes distribution information in clinical diagnosis. AP clustering results are used to classify and select the randomly-generated seeds for ISA, which ensures that the seeds could reflect the subtypes’ incidence. Then different thresholds are set for different seed categories, in order that the biclustering results keep consistent with the real subtypes’ occurrence rate as far as possible.

## Results

Several studies have shown that breast tumors can be divided into at least five molecular subtypes based on gene expression profiles. Indeed, different subtypes have different expression patterns. Luminal/ER+ breast cancer is the most heterogeneous in terms of gene expression and patient outcomes, ~66% of clinically tumors fall into Luminal subtype in the dataset used in this paper. The basal-like tumors are typically negative for ER, PR and HER2, so these tumors are often referred to triple-negative breast cancers (TNBCs). Only ~18% of clinically tumors fall into basal-like subtype. HER2 subtype deals with DNA amplification of HER2 and over-expression of multiple HER2-amplicon-associated genes, and ~11% of tumors are HER2-enriched. The other 5% breast tumors are Normal-like subtype. In this study, we used the PAM50-defined subtype predictor as the classification metric.

AP-ISA algorithm was performed on the dataset for clustering analysis using previously published ‘intrinsic gene list’ [6]. We carried out AP clustering to analyze all samples with the parameter K = 5, since there are five acknowledged subtypes in breast cancer. Although the set size of possible input seeds is huge, there exists a rather limited number of fixed points for given thresholds (*t*_*G*_, *t*_*C*_) [16]. Therefore we set the initial seeds number to 10,000, which is big enough. Then, 10,000 random sparse 0/1 vectors were created with size equal to the samples number. These sparse 0/1 vectors, acting as seeds, were filtered and clustered to five seed types according to the result of AP clustering. For the sake of calculating convenience, 100 seeds were selected randomly based on the ratio of five seed types and applied to ISA algorithm, including 30, 15, 35, 15 and 5 in every seed set. For AP-ISA, the content of a particular module depends on the thresholds (*t*_*G*_, *t*_*C*_). It is noted that there is a hierarchical structure of modules that persists over a finite range of the thresholds. This hierarchical structure resembles the tree structures and have the characteristic that branches may share common genes or conditions. So we try *t*_*G*_ and *t*_*C*_ in the range of [1, 2] and finally, for the five subtypes, *t*_*G*_ was set to 1, 1.4, 0.9, 1.4 and 2 respectively, while *t*_*C*_ was set to 1.6 consistently.

AP-ISA biclustering results highlight many conclusions from the original work of Sørlie et al. [2–4]. Some results are verified by other works [9, 23]. We also achieve some new results that need further investigation. Detailed results are listed as follow.

### Gene expression and clinical analysis

Nine biclusters were obtained by AP-ISA algorithm. Table 1 shows the samples number in nine biclusters based on the label of PAM50-defined subtypes. Figures S1 to S9 in Additional file 1 summarize the composition of each bicluster.

**Table 1:** AP-ISA biclusters composition comparing to PAM-50 labels

	Basal-like	HER2+	LuminalA	LuminalB	Normal-like	Totalnum
Bicluster1	0	0	6	0	24	30
Bicluster2	5	4	9	1	3	22
Bicluster3	0	42	5	8	0	55
Bicluster4	90	1	0	0	0	91
Bicluster5	0	0	33	25	1	59
Bicluster6	0	0	31	17	0	49
Bicluster7	0	3	22	33	1	59
Bicluster8	37	16	22	14	3	92
Bicluster9	0	0	97	19	0	117

The biclustering results exhibit correspondence with PAM50 labels in some degree. Most Normal-like, HER2-enriched and Basal-like samples fall into three different biclusters, that is, Bicluster 1, 3 and 4. Whereas, most Luminal samples split into four biclusters: one luminalA biclusters (Bicluster 9), and the other three biclusters are composed of mixed samples from LuminalA and LuminalB (Biclusters 5, 6 and 7). For Bicluster 2 and 8, We cannot obtain valuable information in enrichment analysis and methylation analysis, which might be due to the fact that they are composed of samples from all the subtypes. Therefore, Bicluster 2 and 8 did not be mentioned in subsequent analysis. Furthermore, we consider ER and PR as classification factor [26, 27].

Basal-like subtype (Bicluster 4) is often referred to triple-negative breast cancer (TNBCs) [28]. ~90% breast tumors are typically negative for ER and PR in AP-ISA biclusters, which are listed in Table 2. Basal-like tumors contain high expression genes that associate with cell proliferation. Detailed gene information is shown in Figure S4 of Additional file 1. AP-ISA biclustering method also identified some over-expressed genes, like ROPN1, CRABP1 [29], MIA and FOXC1 [30, 31]. Given that most Basal-like breast cancers have bad prognosis, finding new drug targets for this group is critical. Our study suggests that these genes or mediation pathway these genes regulated might provide therapeutic targets.

**Table 2:** Sample number of ER and PR status in biclusters from AP-ISA

Class type	ER+	ER-	PR+	PR-
Bicluster 1	27	3	23	6
Bicluster 2	15	6	14	7
Bicluster 3	33	19	23	31
Bicluster 4	11	75	6	79
Bicluster 5	57	1	47	10
Bicluster 6	49	0	45	4
Bicluster 7	57	0	50	7
Bicluster 8	50	40	43	46
Bicluster 9	112	2	108	6

HER2 DNA amplification is a characteristic signature for HER2 breast tumors [32]. Unlike other biclusters, HER2 subtype (Bicluster 3) shows less characteristic in ER status as shown in Table 2. This study also highlights DNA amplification of other potential therapeutic targets in HER2-enriched subtype, including genes FGFR4 [33], TCAP and GRP7 [34].

Luminal breast cancer is the most heterogeneous in terms of gene expression, though they are typically positive for ER and PR as shown in Table 2. In this study, luminal samples were split into four biclusters. We designate them as Luminal-5 (Bicluster5), Luminal-6 (Bicluster6), luminal-7 (Bicluster7) and Luminal-9 (Bicluster9). High mRNA and protein expression in breast luminal cells is one feature of luminal subtype, including genes ESR1, XBP1, GATA3 [35, 36] and MYB. To explore its substructures, we referred PAM50 class labels in Table 1.

The most obvious property of the resulting partitions was different gene composition and expression pattern in each luminal bicluster. Indeed, the four luminal biclusters have different genes and samples. Luminal-9 subgroup, in which totally 93 genes are over-expressed, is composed of samples almost all from LuminalA, and there is only several genes overlapping with the other luminal subgroups. Some LuminalA samples are contained within Luminal-5, Luminal-6 and Luminal-7, which composed of both Luminal A and Luminal B samples. This suggests that Luminal-5, Luminal-6 and Luminal-7 samples are much similar to luminal B samples in expression profile, while compared with samples in Luminal-9.

Genes expression heatmap reveals that Luminal-5 samples are typically over-expressed in PVALB, CGA [37, 38] and TRH. A number of over-expressed genes, like GRIA2 and CYP2A7, are related to Luminal-6. In contrast, Luminal-7 subgroup, which is enriched with LuminalB samples, does not have obvious manifestation comparing to other biclusters. There is no overlapping gene across four biclusters. According to these results, we suggest that Luminal samples can be further partitioned into finer subgroups, which tallies with the recent research [9]. This new subtype partition may have important clinical meaning for breast cancer.

**Fig. 1:**
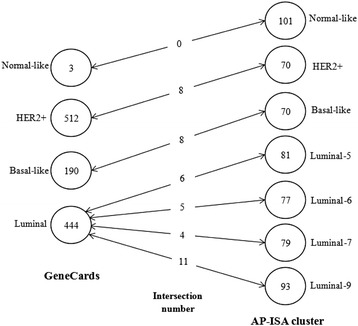
Gene comparsion between biclsutering and GeneCards database. Left side represents the number of genes in GeneCards, right side represents the result of biclsutering in our study, while the middle column stand for intersection number. Four Luminal subgroups in our study all intersect with Luminal in GeneCards

To further validate the effectiveness of AP-ISA, we investigated the genes related to breast cancer subtypes in GeneCards database (http://www.genecards.org/). In this database, there are three genes associated to Normal-like, 190 to Basal-like, 512 to HER2+, and 444 to Luminal subtype respectively. We intersected the genes for each subtype between AP-ISA results and GeneCards database in Fig. 1. Left side of Fig. 1 represents the number of genes in GeneCards, right side represents the AP-ISA result, while the middle column stands for intersection gene number. Four Luminal subgroups in our study intersect with Luminal type in GeneCards.

Table 3 lists the intersection genes in each breast cancer subtype between AP-ISA clusters and GeneCards. In previous analysis, Lumianl-7 did not show obvious pattern in gene expression. However, Luminal-7 has 4 overlapping genes with genes associated with Luminal subtype in GeneCards database. Furthermore, almost all intersection genes in Table 3 are mentioned in previous analysis, like GRB7, ERBB2 in HER2+, FABP7 in Basal-like, ESR1, XBP1 in Luminal. In summary, many genes in AP-ISA results consist with currently acknowledged genes, which proves the accuracy and reliability of AP-ISA for classification of breast cancer.

**Table 3:** Intersection genes between AP-ISA biclusters and GeneCards database

Subtype	Intersection gene number	Genes
HER2+	8	GRB7;ERBB2;CASP3;SDC1;STARD3;ABCC3; GSK3B;CEACAM5
Basal-like	8	GABRP;MSH2;CDKN2A;EN1;YBX1;VGLL1; FABP7; FOXC1
Luminal-5	6	BCL2;GATA3;RERG;ESR1; BAG1;CCND1
Luminal-6	5	BCL2;ESR1;DACH1;BAG1;XBP1
Luminal-7	4	SLC9A3R1;KRT19;CANX;YWHAZ
Luminal-9	11	PGR;EPHX2;BCL2;CYB5A;MUC1;RAB31;MYB; ESR1;SREBF1;XBP1;LRIG1

### Enrichment analysis

In order to identify the genes that can distinguish breast cancer subtypes, we performed Gene Ontology and KEGG Pathways enrichment analysis, according to the subtype partition achieved by AP-ISA. Analysis results are shown in Table 4.

**Table 4:** Significant genes in AP-ISA biclusters and the most distinct gene enrichment pathways by Gene Ontology and KEGG

Class type	Term (Enrichiment type)	*P*-value	significant genes
Normal-like	regulation of cell proliferation (Gene Ontology)	4.38E-10	CDKN1C;TXNIP;DPT;EDNRB; KL; FIGF;ANXA1;NRG1;HOXA5; ID4; ID4;IGF1;IGFBP6;AQP1;KIT;AQP1; LIFR;PPARG;PRNP;NDRG2; CAV1 PTN;PTPRM;RBP4;CX3CL1; CAV2; SFRP1;TGFBR2;TGFBR3; KLF4; KRT5;PPAP2B;KRT17;CD36; RBP4;
	regulation of multicellular organismal process (Gene Ontology)	1.03E-09	
	cell differentiation (Gene Ontology)	1.07E-08	
	PPAR signaling pathway (KEGG Pathways)	4.58E-04	
HER2+	single-organism process	1.29E-04	ERBB2;FGFR4;GRB7;GSK3B; FA2H; PSMD3;BIK;CDC6;CLTC;GSK3B; ODF2;RAP1GAP;S100A8;SDC1;CDC6; STX1A;TMSB10;SNF8;FHOD1; EAF2;VPS37B;WIPF2;TCAP;STARD3;
	epidermal growth factor receptor signaling pathway (Gene Ontology)	6.944E-03	
	ERBB signaling pathway (Gene Ontology)	7.514E-03	
Basal-like	cell cycle process	1.09E-05	CDK6;CDKN2A;MSH2;FZD9; FABP7; LY6D;BCL11A;CCNE1;CDC20; MIA; CDK6;CDKN2A;CENPA;FANCA; FOXC1;STMN1;MSH2;TTK;EN1; CDK2AP1;RAD54L;CDC123;DSC2; GTPBP4;PHGDH;CDCA8;B3GNT5; CENPN;TTYH1;SUV39H2;ROPN1; CRABP1;KLK6; VGLL1;SERPINB5;
	lymphocyte differentiation (Gene Ontology)	1.019E-03	
	B cell activation (Gene Ontology)	8.673E-03	
	p53 signaling pathway (KEGG Pathways)	3.15E-04	
	Pathways in cancer (KEGG Pathways)	4.489E-03	
Luminal-5	mammary gland epithelium development	1.15E-05	CCND1;ESR1;GATA3;TBX3; BTF3; WNT3;BCL2;CELSR2;TLE3;CGA; RNF43;PVALB;CPB1;SLC1A2; SKP1A; C5orf30;SLC16A6;BEX1;GLDC;HAGH; ZNF24;LRBA;C6orf211;YPEL3;COX6C; LAMA3;MKL2;RAD17;BCAS1; CGN; SERPINA5;HSPB8;COX17;ING2;
	Wnt signaling pathway (Gene Ontology)	7.896E-03	
	CD8-positive, alpha-beta T cell lineage commitmen (Gene Ontology)	4.294E-03	
Luminal-6	Glutamate receptor signaling pathway (Gene Ontology)	2.316E-03	BCL2;WNT3;ESR1;SERP1;PIGT; TLE3;STC1;ARNT2;PKIB;ZFX; HAGH; IGBP1;HPN;DNAJC12;TBCA;BCAS1; CCNH;ACBD4;GRIA2;CYP2A7;BAI2; GRIA1;XBP1;SIAH2;CPEB4; MAP2K4; SLC27A2;PNPLA4;SLC1A2; MAST4; CYB5R1;CARTPT;RABEP1;RAD17; COX6C;QDPR;SEC11C;
	CD8-positive, alpha-beta T cell lineage commitment (Gene Ontology)	3.87E-03	
	response to insulin-like growth factor stimulus (Gene Ontology)	7.726E-03	
	Retinol metabolism (KEGG Pathways)	4.07E-03	
Luminal-9	CD8-positive, alpha-beta T cell lineage commitment (Gene Ontology)	4.717E-03	XBP1;BCL2;C3orf18;CIRBP;GAD1; PKIB;APH1B;NAT1;RAB30; ABAT; BCL2;MYO5C;CA12;SIAH2;MKL2; TTC12;REPS2;NPY1R;KIAA1370; NAT2;RALGPS2;CYBRD1;MUC1; RAB31;RLN2;NTN4;MAP2K4; MAST4;GALNT10;MYB;ESR1; SREBF1;GFALS;TLE3;XBP1; ACBD4;STC2;ABAT;
	response to insulin-like growth factor stimulus (Gene Ontology)	9.412E-03	
	beta-Alanine metabolism (KEGG Pathways)	5.476E-03	

It is observed that, the two genes KRT17 and KRT5, which gathered in bicluster 1, are over-expressed in breast basal epithelial cells of Normal-like samples. Regulating genes about cell proliferation and cell differentiation appeared in Normal-like subtype. This fact is based on two annotations (Gene Ontology: “regulation of cell proliferation” *p* = 4.38E-10, Gene Ontology: “cell differentiation” *p* = 1.07E-08). We also find KEGG Pathways “PPAR signaling pathway” (*p* = 4.58E-04) in this subtype [39].

**Fig. 2:**
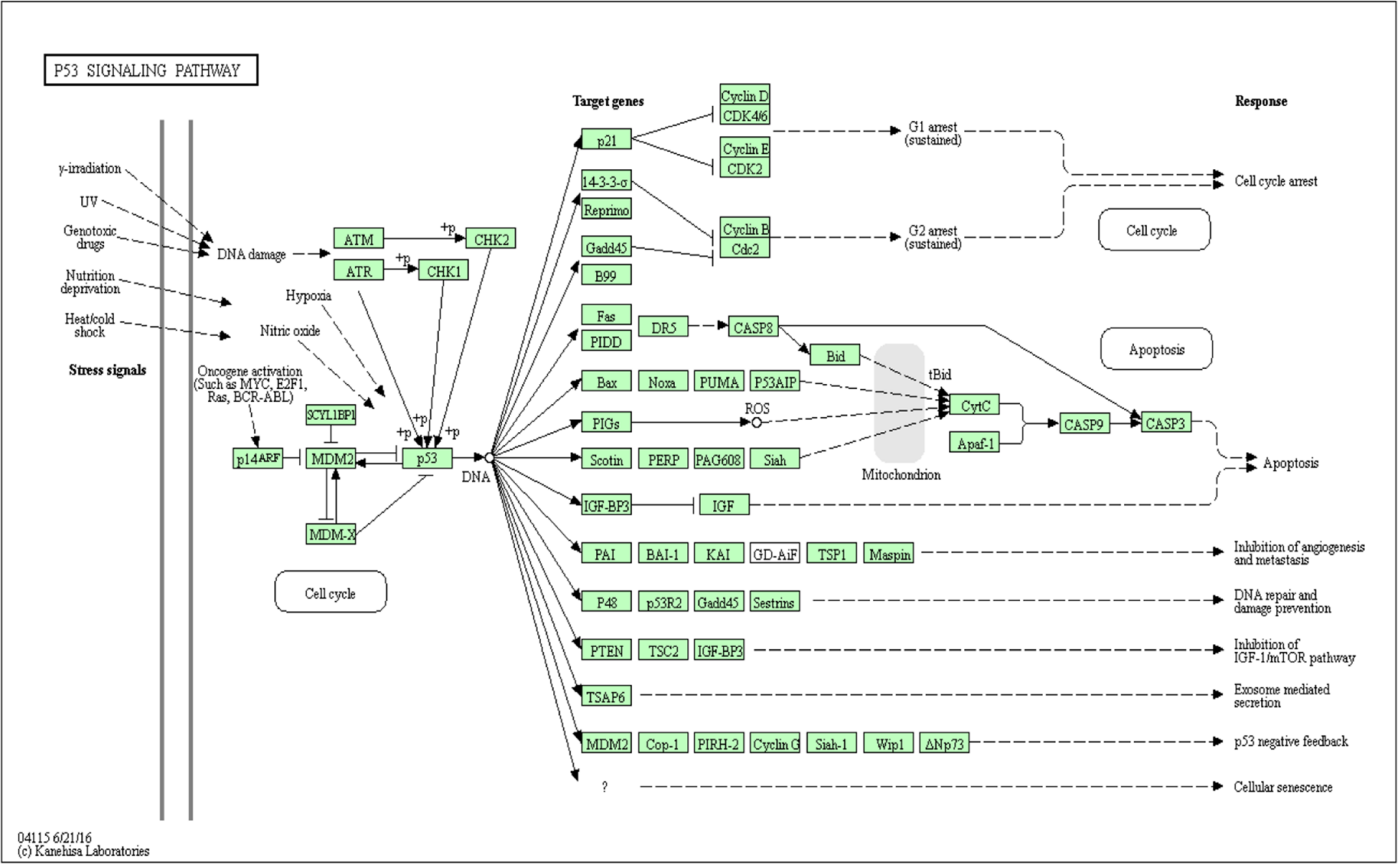
Over-expressed genes of Basal-like samples in p53 signaling pathway. Some over-expressed genes in Basal-like were found to be significantly enriched for the pathway genes (*p* = 3.15E-05). Pathway and graphics were taken from the Kyoto Encyclopedia of Genes and Genomes (KEGG) database

HER2-enriched samples, which are mostly gathered in Bicluster 3, exhibit high expression of ERBB2、FGFR4 and GRP7. They play a crucial role in epidermal growth factor receptor signaling pathway (Gene Ontology:“epidermal growth factor receptor signaling pathway” *p* = 6.944E-03) [40]. A number of over-expressed genes in Basal-like samples are related to KEGG Pathways “p53 signaling pathway” (*p* = 3.15E-05, shown in Fig. 2) [41] and “Pathways in cancer” (*p* = 4.489E-03).

For Luminal subtype, on the basis of Gene Ontology, Luminal-5, 6, 9 are typically enriched in “CD8+, alpha-beta T cell lineage commitment” (*p* < 0.5E-02), and “Wnt signaling pathway” [42] (*p* = 7.896E-03) also enriched in Luminal-5. Referring to Lumianl-5, the over-expressed genes in Luminal-6 were related to Retinol metabolism (*p* = 4.07E-03). Gene Ontology “beta-Alanine metabolism” (*p* = 5.476E-03) appeared in Luminal-9. Table 4 contains a list of significant pathways, and the full list can refer to Additional file 2. In summary, samples in each AP-ISA bicluster exhibit significant difference based on the annotation databases.

### Analysis of DNA methylation in AP-ISA biclusters

Breast cancer have been proved to be heterogeneous in gene expression. To further identify and characterize clinically significant markers within breast cancer subtypes, we explored breast cancer patient variability on the epigenetic level as well, using HumanMethylation27 (HM27) and Human Methylation450 (HM450) array dataset that are available from TCGA.

**Fig. 3:**
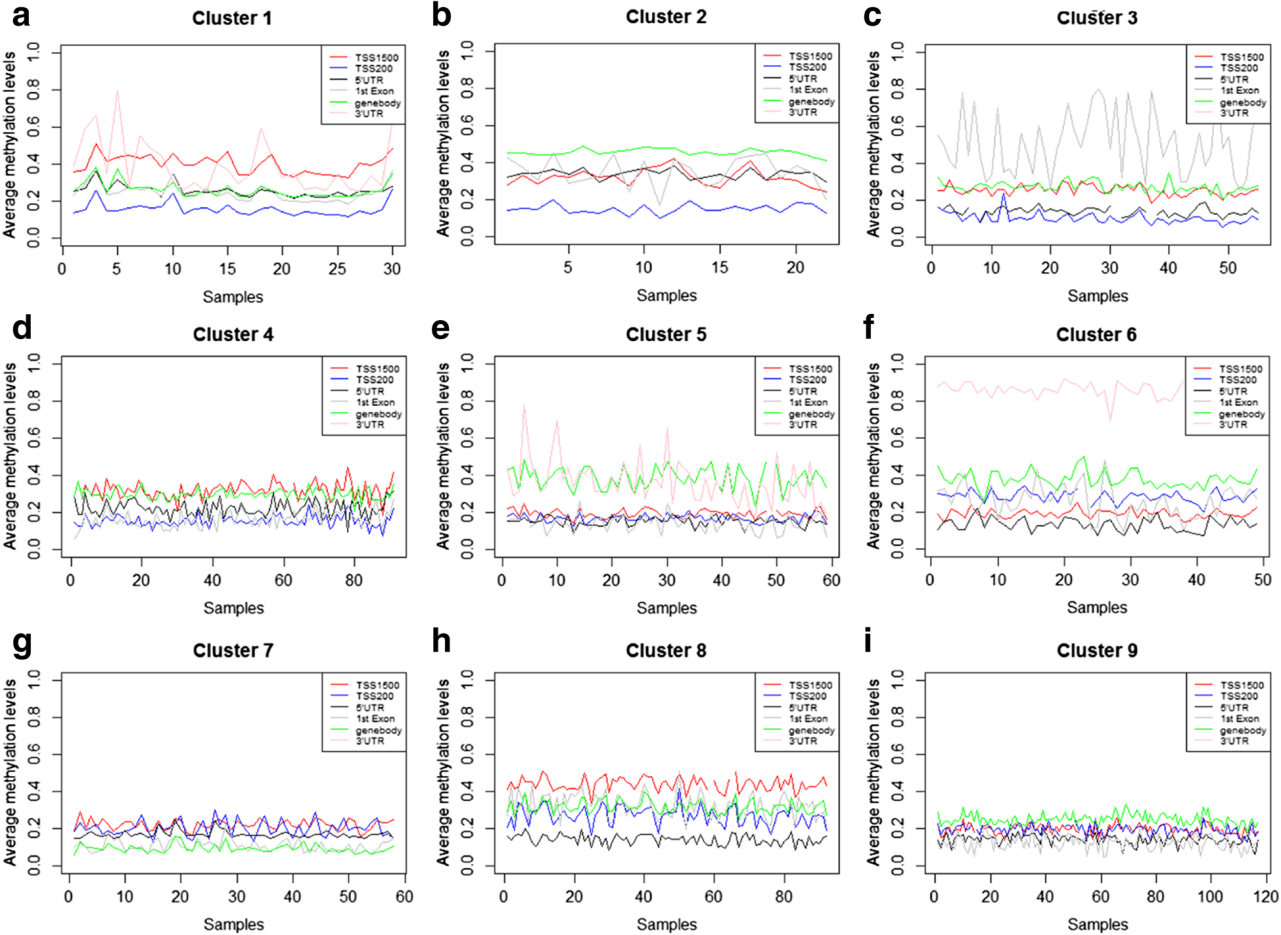
Methylation analysis in six methylation areas exhibits differential methylation level among biclusters. The blue lines, red lines, black and gray lines respectively display TSS200, TSS1500, 5’UTR and 1st Exon area which represent promoter region. The green lines represent genebody and the pink lines 3’UTR. Horizontal axis indicates samples in AP-ISA biclusters. Values of vertical axis were calculated by averaging the methylaiton values in the same sample

In this study, methylation sites were divided into six categories using FEM package in R, including TSS200, TSS1500, 5’UTR, 3’UTR, gene body and 1st Exon [43]. TSS200, TSS1500, 5’UTR and 1st Exon are located in gene promoter region. Considering different gene expression profile in AP-ISA biclusters, we analyze methylation level for different area in each bicluster. Methylation level was measured using average *β* value of CpG sites in the same area for the same sample. Figure 3 shows DNA methylation levels in different area of each bicluster. We focus on TSS200, TSS1500, 5’UTR and gene body, since TSS200, TSS1500 and 5’UTR are near to transcriptional start site (TSS). The situation of gene transcription from TSS directly affects gene expression. For 3’UTR and 1st Exon, AP-ISA results show that, their methylation values fluctuate drastically in some biclusters, such as bicluster1 (Fig. 3a). In other biclusters, no methylation site in 3’UTR and 1^st^ Exon, like bicluster4 (Fig. 3d).

In general, gene body area showed higher methylation level than that in TSS200 and 5’UTR, which are near to TSS, except for Lumianl-7 (Bicluster 7). Normal-like subtype (Fig. 3a) exhibits hypomethylation in TSS200, while hypermethylaion dominates in gene body, 5’UTR and TSS1500, especially in TSS1500. This is similar to methylation level in normal samples.

Referring to Normal-like samples, HER2-enriched subtype samples (Fig. 3c) exhibit a distinct hypomethylation in TSS200, TSS1500 and 5’UTR, which may be associated with DNA amplification of HER2 and over-expression of multiple HER2-amplicon-associated genes. Likewise, all Basal-like samples (Fig. 3d) show hypomethylation in promoter region (TSS200, TSS1500 and 5’UTR).

Most luminal samples were assigned to four different AP-ISA biclusters, that is, Luminal-5, 6, 7, 9. All these samples exhibited hypomethylation in TSS1500, TSS200 and 5’UTR, when compared to Normal-like samples. Luminal-5 (Fig. 3e) and Luminal-6 (Fig. 3f) samples presented hypermethylation in gene body, especially Luminal-6 showed even higher methylation level, while compared to other luminal samples. Luminal-7 (Fig. 3g) and Luminal-9 (Fig. 3j), on the other hand, manifested opposite characteristic. They have lower methylation level in gene body, especially Lumianl-7 samples. In particular, Luminal-6 exhibited up-regulation in TSS200 methylation area, which may be associated with gene silence.

TSS200, TSS1500 and 5’UTR are all in promoter region, but methylation level among them showed difference. In TSS200 and 5’UTR, methylation level is similar, but TSS1500 presents distinction. This observation mainly highlights in HER2+ (Fig. 3c) and Basal-like subtype (Fig. 3d). In Luminal-5, Luminal-7 and Luminal-9 subgroups, the methylation patterns are consistent. In conclusion, HER2-enriched and Basal-like subtype exhibited hypomethylaion in promoter region, which related to up-regulation in related genes. For Luminal subtypes, low methylation level existed in LuminalB-enriched Luminal-7 and LuminalA-enriched Luminal-9, between which difference are significant in the gene body. Luminal-5 showed similar methylation levels in TSS200, 5’UTR and gene body comparing to HER2-enriched and Basal-like, suggesting that the methylation pattern of Luminal-5 is closer to HER2-enriched and Basal-like. Thus, each breast cancer subtype has its distinct methylation pattern. Noting that, although TSS200, TSS1500 and 5’UTR are all located in promoter region, their methylation level are different obviously.

There is no apparent methylation pattern in bicluster 2 (Fig. 3b) and 8 (Fig. 3h), since methylation values fluctuate drastically. Experimental results show that different breast cancer subtype has different methylation pattern, and gene expression is related to methylation in subtypes. We suggest that DNA methylation should be taken into account in breast cancer remedy, together with subtype information.

## Algorithm comparison and validation

AP-ISA is based on ISA [14]. Besides ISA, there are several state-of-the-art biclustering methods, such as Large Average Submatrices (LAS) [44], The Cheng and Church biclustering algorithm (CC) [11], Sparse Biclustering (Sparse BC) [45] and Sparse Singular Value Decomposition (SSVD) [46]. We compare AP-ISA with these methods.

LAS, CC and SSVD allow users to choose the number of generated biclusters. We set 10 biclusters for the three method, to compare with the result of AP-ISA, from which we obtained nine biclusters. We set *δ* = 0.1 For CC, Score cut off as 1000 for LAS to find the biclusters higher than the score cut off. SSVD initially ran with the parameter *gamu* = *gamv* = 2 according to the reference [46], but it produced biclusters that contained most of the available genes and samples. To solve this problem, we increased *gamu* and *gamv* from 2 to 30. The settings of sparese BC were *K* = *R* = 10, and *λ* is calculated by BIC, in order to guarantee that the result is comparable to the other methods. In ISA, the row and column thresholds were set to 1.6. We analyze these methods from three aspects and the comparison results are shown as follows.

### Bicluster size

**Fig. 4:**
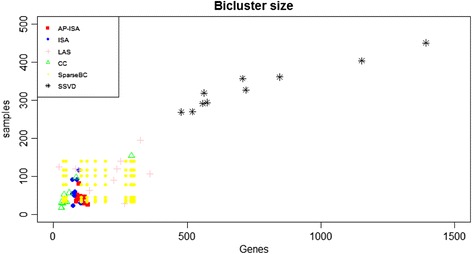
Bicluster size

Figure 4 shows the row and column dimensions of the biclusters produced by all the methods. LAS and CC generate a relatively wide range of biclsuter sizes, with those of LAS from 21 to 361 in gene and from 62 to195 in sample. Biclusters obtained by SSVD have large number of samples and genes, with more than 260 samples and 500 genes in every case. Noting that, the number of biclusters produced by Sparse Biclustering is *K* × *R*, ranging from 32 × 37 to 139 × 297, while the size range of ISA’s biclusters are small. By contrast, AP-ISA’s biclsuters are with moderate size and the number of samples are neither too small nor too big.

### Effective number of biclusters

Most biclustering algorithms allow to overlapped members among biclusters. The favorable side is that overlapped gene and sample sets can capture underlying biological mechanism, where a gene may play role in multiple biological pathways or other activities. However, too much overlap may reduce the effective output. For example, two biclusters with high overlapping rate do not provide much more information than either bicluster [44]. We use function *F*(∙) to measure the effective number of biclusters in *U*_1_, ⋯, *U*_*K*_ by the following equation [44]:

$$ F\left({U}_1,,\cdots, {U}_K\right)=\sum \limits_{k=1}^K\left(\frac{1}{\left|{U}_K\right|}{\sum}_{x\in {U}_K}\frac{1}{N(x)}\right) $$

In the above equation, $N(x)=\sum \limits_{k=1}^K1\left\{x\in \left.{U}_K\right\}\right.$ is the number of biclusters containing matrix entry *x*, 1/*N*(*x*) means the contribution that the element *x* made to biclsuter *U*_*K*_. For example, for a entry *x* in *U*_*K*_, the contribution to *U*_*K*_ is 1, if *x* exists only in group *U*_*K*_. Otherwise, the contribution to *U*_*K*_ is 1/*p*, if *p* biclusters contain entry *x*. *F*(∙) has the property that if, for any 1 ≤ *r* ≤ *K*, the biclusters *U*_1_, ⋯, *U*_*K*_ can be divided into *r* non-overlapping groups of identical biclusters, then *F*(*U*_1_, ⋯, *U*_*K*_) = *r*.

Table 5 shows the effective number of biclusters generated by the biclustering methods. The low overlap of CC originates from the fact that it replaces missing data in the matrices with random numbers. The low overlap of Sparse Biclustering is due to the fact that it is actually an extending sparse one-way clustering and it assumed that each observation and feature belong to an unknown and non-overlapping classes respectively. The high overlap of SSVD is explained in part by their large size. Biclusters obtained by AP-ISA have moderate levels of overlap, less than other methods, except CC and Sparse Biclustering.

**Table 5:** Comparison of total number of biclusters, effective number of biclusters and the ratio of the effective number to the total number of biclusters

Method	Total number of biclsuters	Eff. number of biclusters	Ratio
AP-ISA	9	6.743	0.749
ISA	12	8.489	0.707
LAS	10	4.799	0.479
CC	10	10	1
Sparse BC	70	70	1
SSVD	10	1.57	0.157

### Subtype capture

The aim of our study is to find breast cancer subtypes and its related genes. We have obtained breast cancer subtypes by AP-ISA, and compared it with PAM50. Here we compare the ability of capturing subtype samples based on PAM50. For each method, we identified the biclusters that matched each subtype in PAM50. Table 6 lists the results.

**Table 6:** Biclusters in each method that match with PAM50

PAM50	AP-ISA	ISA	LAS	CC	SparseBC	SSVD
Basal-like	4	5	1, 10	5, 7	3	–
ERBB2+	3	6	8	–	–	–
LuminalA	5, 6, 9	1, 4	2, 3, 7	1	4, 6	–
LuminalB	5, 7	–	3	–	1, 4	–
Normal-like	1	2	2, 6	1	7	–

We pick out the biclusters which can obviously reflect subtypes, that is, samples in the bicluster has high overlapping rate with a subtype in PAM50. SSVD cannot work, since its biclusters have large size and consist all subtype samples in PAM50. For LAS, the biclusters can match with PAM50 subtype. However, some biclusters are mixture of different subtypes. For example, bicluster 2 in LAS contains Normal-like and Luminal samples, which are significantly different. Bicluster 5 and 7 in CC identified Basal-like samples, but the samples’ number is too small to reflect the Basal-like subtype truly. LuminalB in ISA and CC, ERBB2+ in CC and Sparse Biclustering have not been captured. The information in Table 6 exhibits that AP-ISA is an effective method to capture breast cancer subtypes and it can not only capture each subtype, but also distinguish subtypes much accurately than PAM50.

## Discussion

Gene expression profiling has been proved to be useful for breast cancer classification and treatment. In previous studies, unsupervised clustering, like hierarchical clustering, was performed on breast cancer samples. These methods can only find the global patterns in gene expression profiles. In order to discover subtype-related patterns, we proposed and applied a modified ISA biclustering algorithm, AP-ISA, on breast cancer gene expression profiles to reveal new genetic patterns. Biclustering method allows to cluster subset of patients and genes simultaneously. In AP-ISA, AP clustering was carried out before ISA biclustering to select seeds as prior knowledge, and different thresholds were set for different seeds. This process results in different bicluster size comparing to ISA with randomly selected seeds and the same threshold, which is better to explain breast cancer subtypes in clinical diagnosis.

HER2-enriched samples (bicluster 3) and the Basal-like samples (bicluster 4) conform to PAM50 labels to a great extent. HER2-enrichied subtype exhibits up-regulation in ERBB2, GRP7 and some other genes, such as FGFR4 and TCAP. Enrichment analysis shows that HER2-enrichied subtype is associated with epidermal growth factor receptor signaling pathway (GO:0007173 *p* = 6.944E-03). Activation of tyrosine kinase receptors from the human epidermal growth factor receptor family, related with gene EGFR, HER2, HER3, HER4, plays a key role in the initiation and progression of breast cancer [38]. Anti-HER2 is a validated therapeutic treatment, as shown by the clinical efficacy of trastuzumab and lapatinib.

Genes in Basal-like subgroup, which is ER-negative, PR-negative and HER2-negative, are enriched in p53 signaling pathway (KEGG: 4115, *p* = 3.15E-04). Some genes in Basal-like are outstanding in this pathway, like CCNE1, CDK6, CDKN2A, SERPINB5. P53 encodes a tumor suppressor protein containing transcriptional activation, DNA binding, and oligomerization domains. In some study, Basal-like tumors showed a high frequency of p53 mutations [19], which may loss of p53 function combined with p53 signaling pathway activity. This may explain the question that why Basal-like samples have much worse clinical outcomes than other subtypes.

In this study, experimental analysis shows that current separation between luminal A and luminal B is not clear. AP-ISA split the luminal samples into four subgroups: Luminal-5, 6, 7 and 9. Luminal-7, which is enriched with luminalB samples, exhibits a distinct methylation pattern compared to the other three biclsuters, such as gene body shows obvious hypomethylation. In Luminal-5, 6 and 9, genes are significantly enriched in functions related to the immune system, including enrichment of CD8+, alpha-beta T cell lineage commitment (*p* < 0.5E-02). However, this is not apparent in Luminal-7, suggesting that T cell activation processes may play a important role in luminal A patients and give rise to a better outcome than luminal B patients. Among luminalA-enriched biclusters, the methylation pattern of Luminal-5 is closer to HER2+ and Basal-like. Luminal-9, which is mainly composed of LuminalA samples, exhibits hypo-methylation in all areas. Within Lumianl-6 subtype, methylation levels in TSS200 area are higher than the other biclusters. In brief, methylation pattern in each bicluster was different, which may be associated with different expression patterns.

Finally, we evaluated AP-ISA and five state-of-the-art biclustering methods using a variety of quantitative and biological validation measures. The biclusters generated by AP-ISA present moderate sample size and low overlapping rate. These features implies that AP-ISA can capture disease subtypes across appropriate range of different scales and distinct them accurately. Furthermore, AP-ISA outperforms other methods in capturing breast cancer subtypes.

## Conclusions

This study used a novel biclustering algorithm AP-ISA to classify breast cancer into seven subtypes. For Normal-like, HER2-enriched and Basal-like samples, AP-ISA agrees with PAM50 calls’, while for luminal samples, AP-ISA obtains better performance. LuminalB-enriched Luminal-7 bicluster exhibits lower immune processing and methylation levels, this may be associated with bad prognosis. Luminal-5 is closer to HER2+ and Basal-like subtype. Besides published genes in breast cancer subtypes, we obtain some new genes that would be useful for targeted treatments of breast cancer. AP-ISA is compared with some state-of-the-art methods from bicluster size, effective number of biclusters and subtype capture capability. It is shown that, our study improves the existing methods, and achieves more accurate subgroups, which can contribute to the development of novel directed therapies. Further research is needed in order to consolidate the novel partitions identified in this paper, using survival analysis or other prognostic and diagnostic means in clinical operation.

## Additional files

Additional file 1: The compressed file includes nine heatmap figures for the nine biclusters obtained by AP-ISA. (ZIP 1803 kb)

Additional file 2: The GO and KEGG enrichment analysis results for the nine AP-ISA biclusters are listed respectively. (ZIP 88 kb)

## Abbreviations

AP: Affinity Propagation
AP-ISA: Iterative Signature Algorithm based on Affinity Propagation
CpG: Cytosine & Phosphoric acid & Guanine
DNA: DeoxyriboNucleic Acid
ISA: Iterative signature algorithm
TCGA: The Cancer Genome Atlas
