# Supplementary figures and images for: Analysis of breast cancer subtypes by AP-ISA biclustering
Source: BMC Bioinformatics. 2017 Nov 14;18:481. doi: 10.1186/s12859-017-1926-z (PMC5686903; doi:10.1186/s12859-017-1926-z)

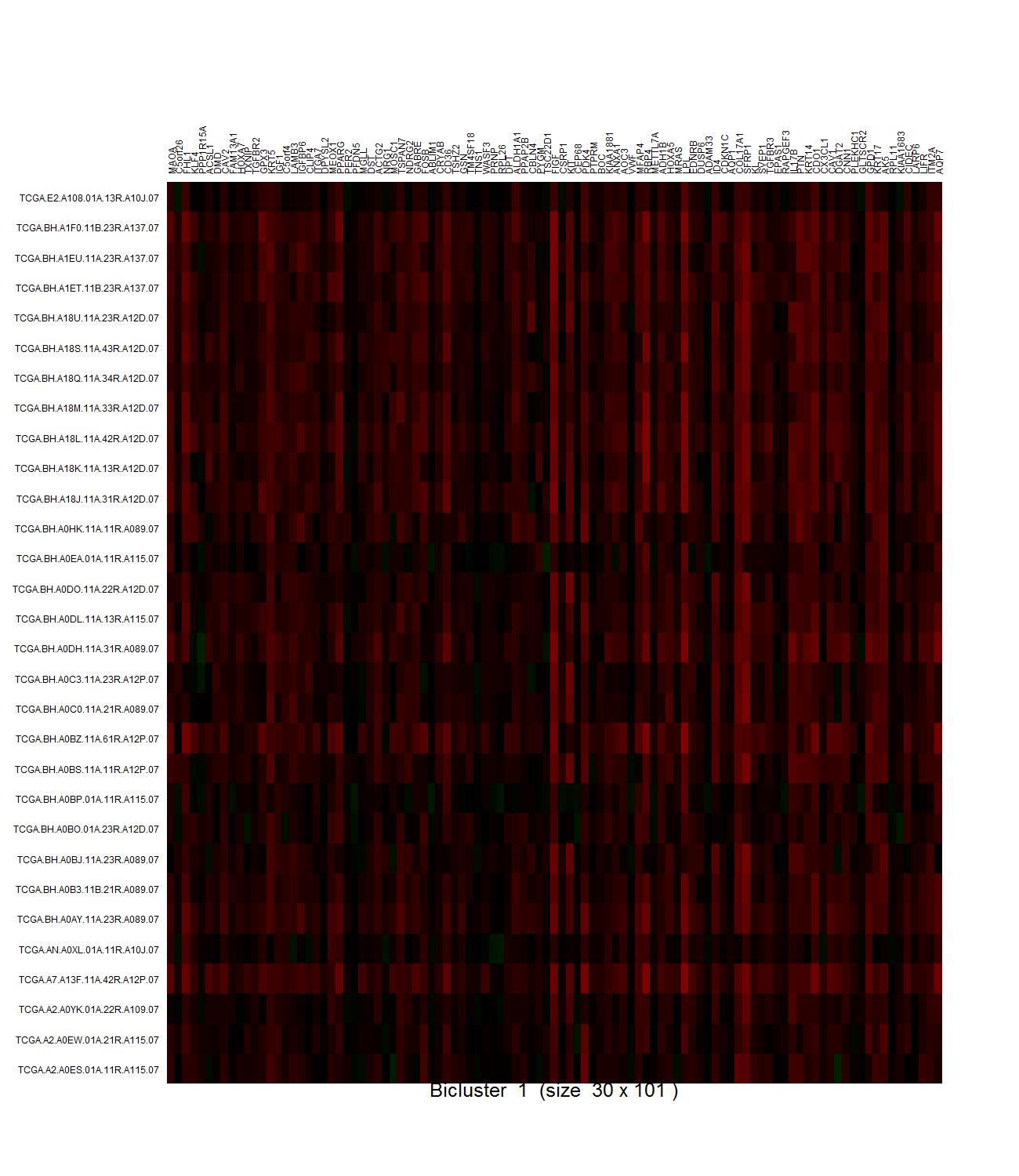

Supplement: Supplementary file 1 — The compressed file includes nine heatmap figures for the nine biclusters obtained by AP-ISA. (ZIP 1803 kb) [file 12859_2017_1926_MOESM1_ESM.zip › heatmap Figure 1-9/Figure S1.jpg]

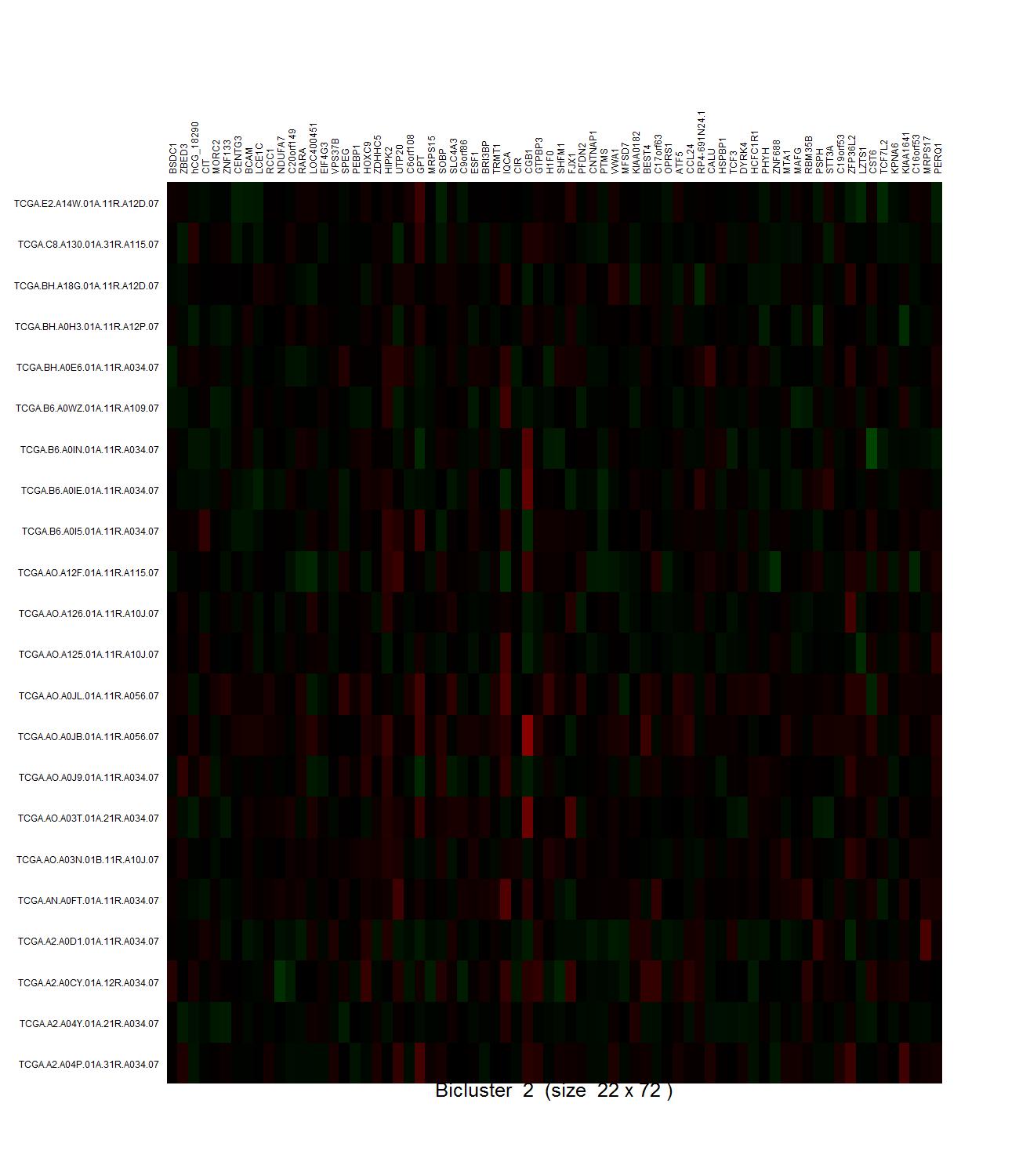

Supplement: Supplementary file 1 — The compressed file includes nine heatmap figures for the nine biclusters obtained by AP-ISA. (ZIP 1803 kb) [file 12859_2017_1926_MOESM1_ESM.zip › heatmap Figure 1-9/Figure S2.jpg]

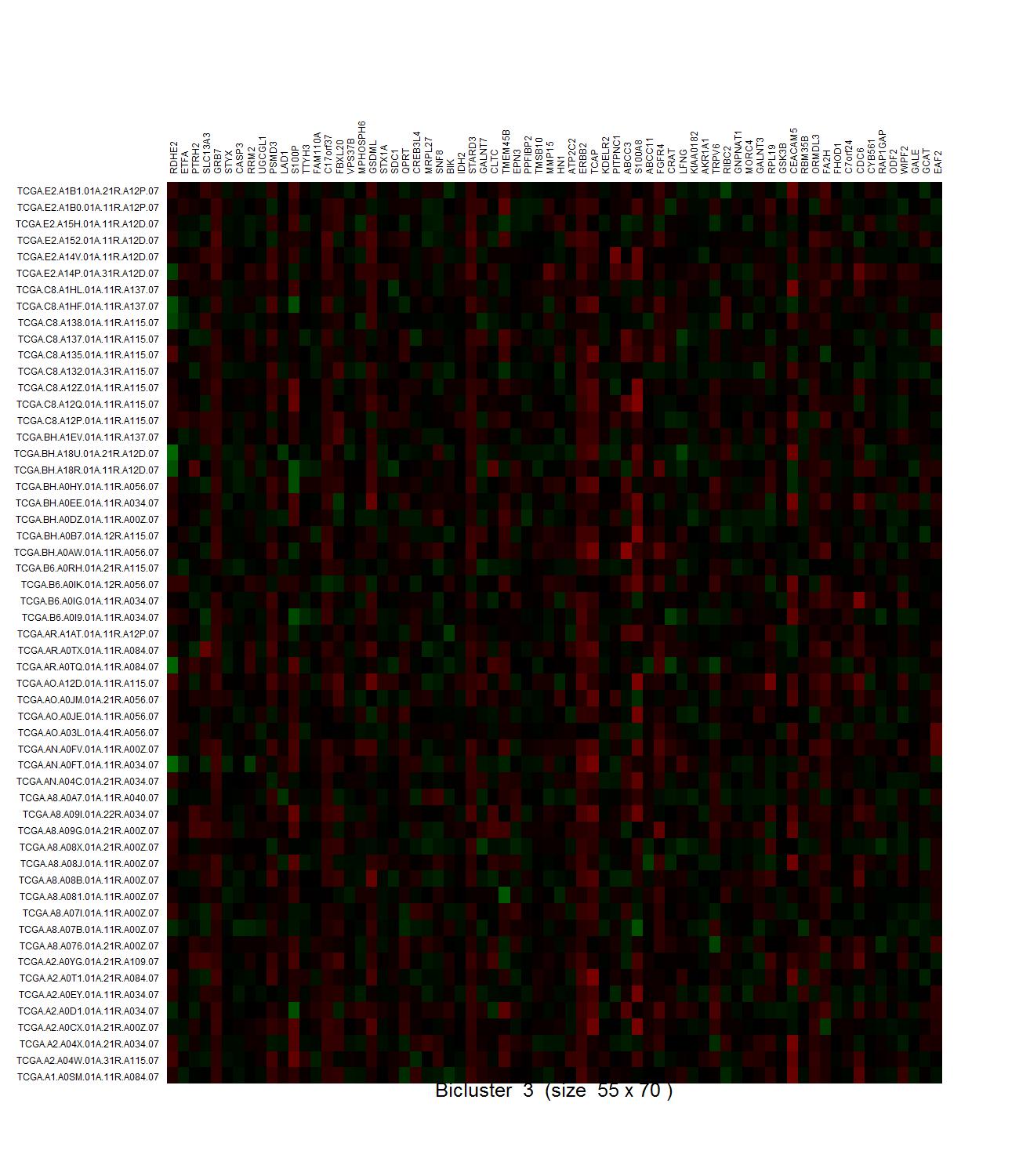

Supplement: Supplementary file 1 — The compressed file includes nine heatmap figures for the nine biclusters obtained by AP-ISA. (ZIP 1803 kb) [file 12859_2017_1926_MOESM1_ESM.zip › heatmap Figure 1-9/Figure S3.jpg]

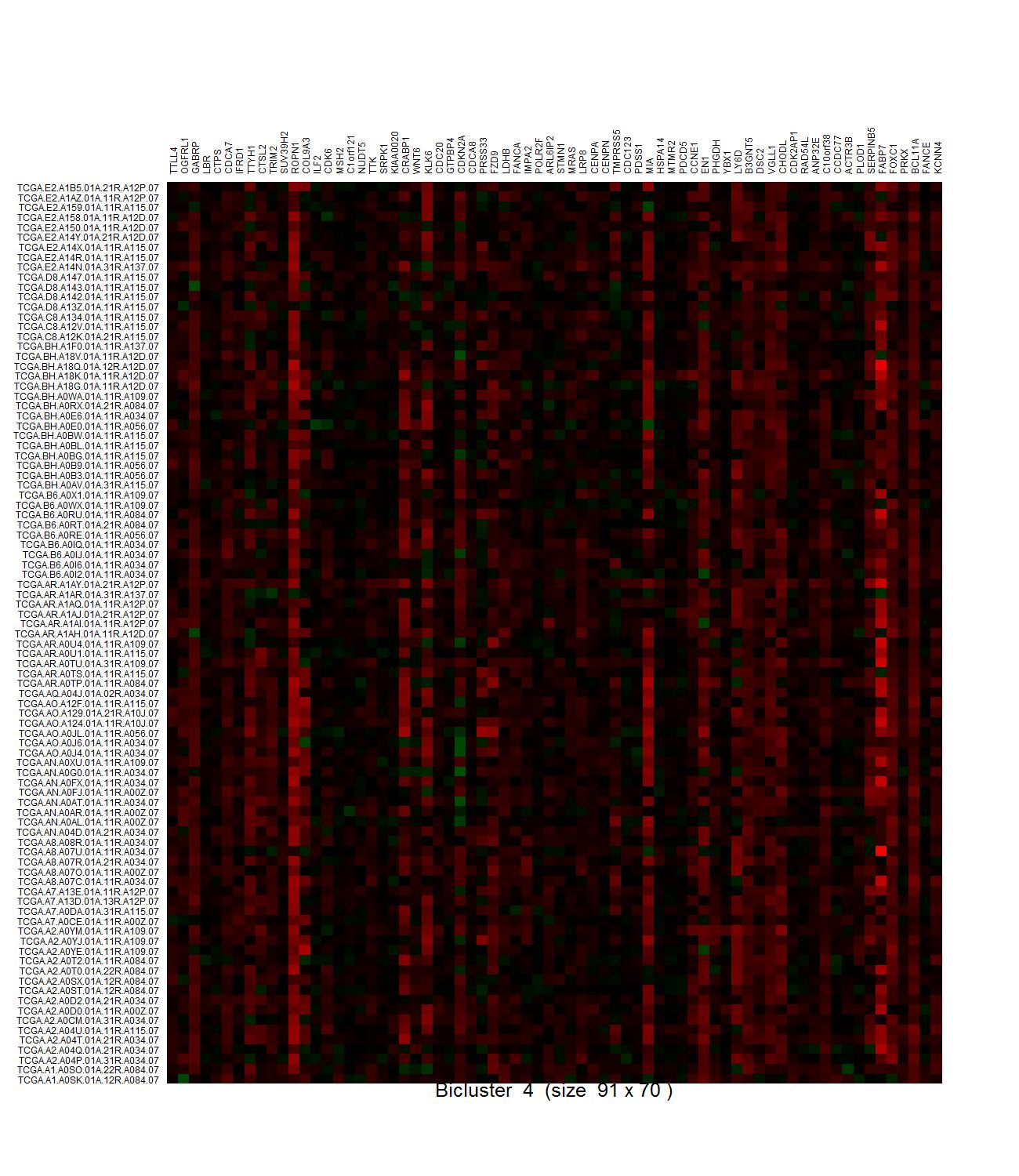

Supplement: Supplementary file 1 — The compressed file includes nine heatmap figures for the nine biclusters obtained by AP-ISA. (ZIP 1803 kb) [file 12859_2017_1926_MOESM1_ESM.zip › heatmap Figure 1-9/Figure S4.jpg]

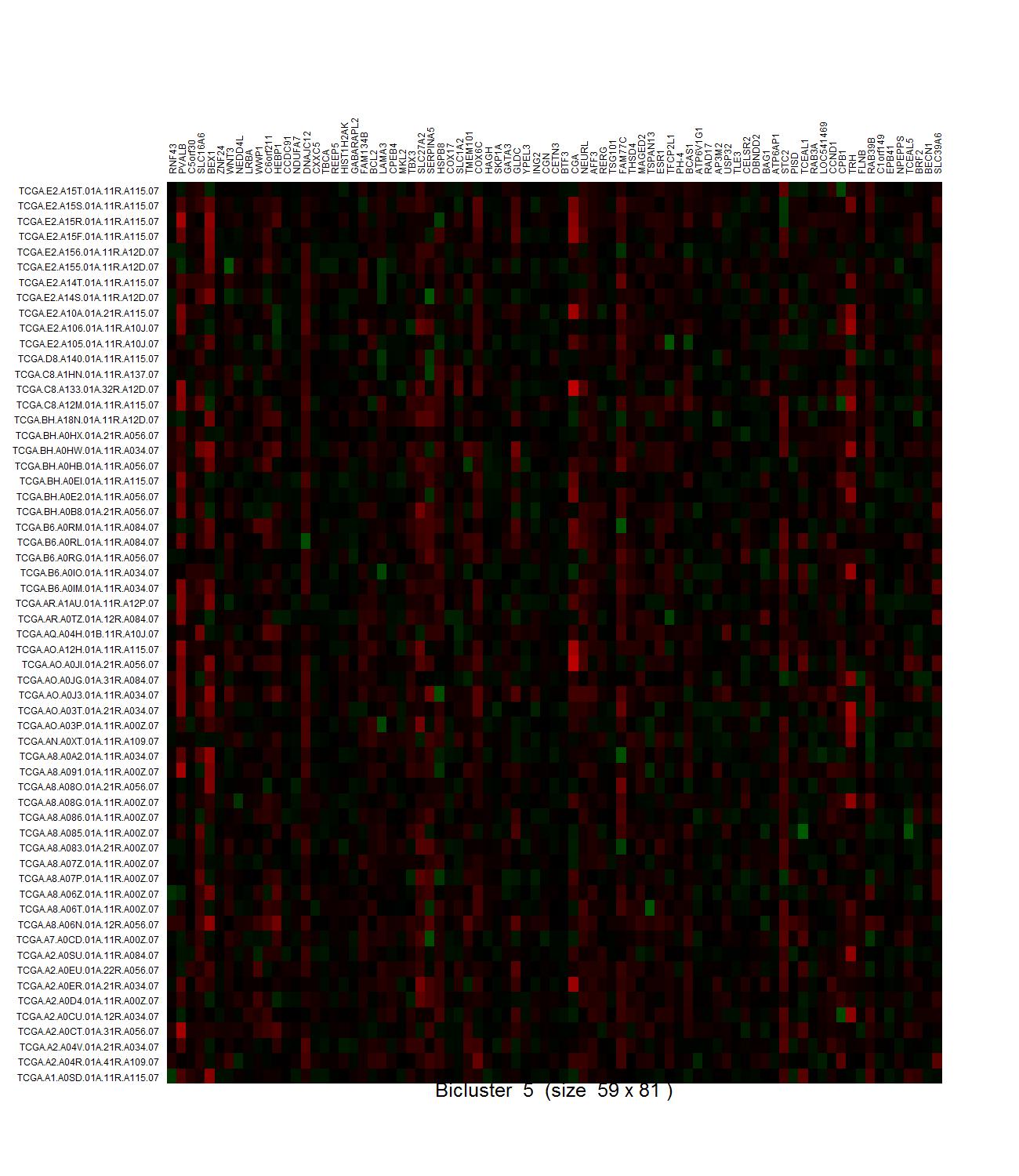

Supplement: Supplementary file 1 — The compressed file includes nine heatmap figures for the nine biclusters obtained by AP-ISA. (ZIP 1803 kb) [file 12859_2017_1926_MOESM1_ESM.zip › heatmap Figure 1-9/Figure S5.jpg]

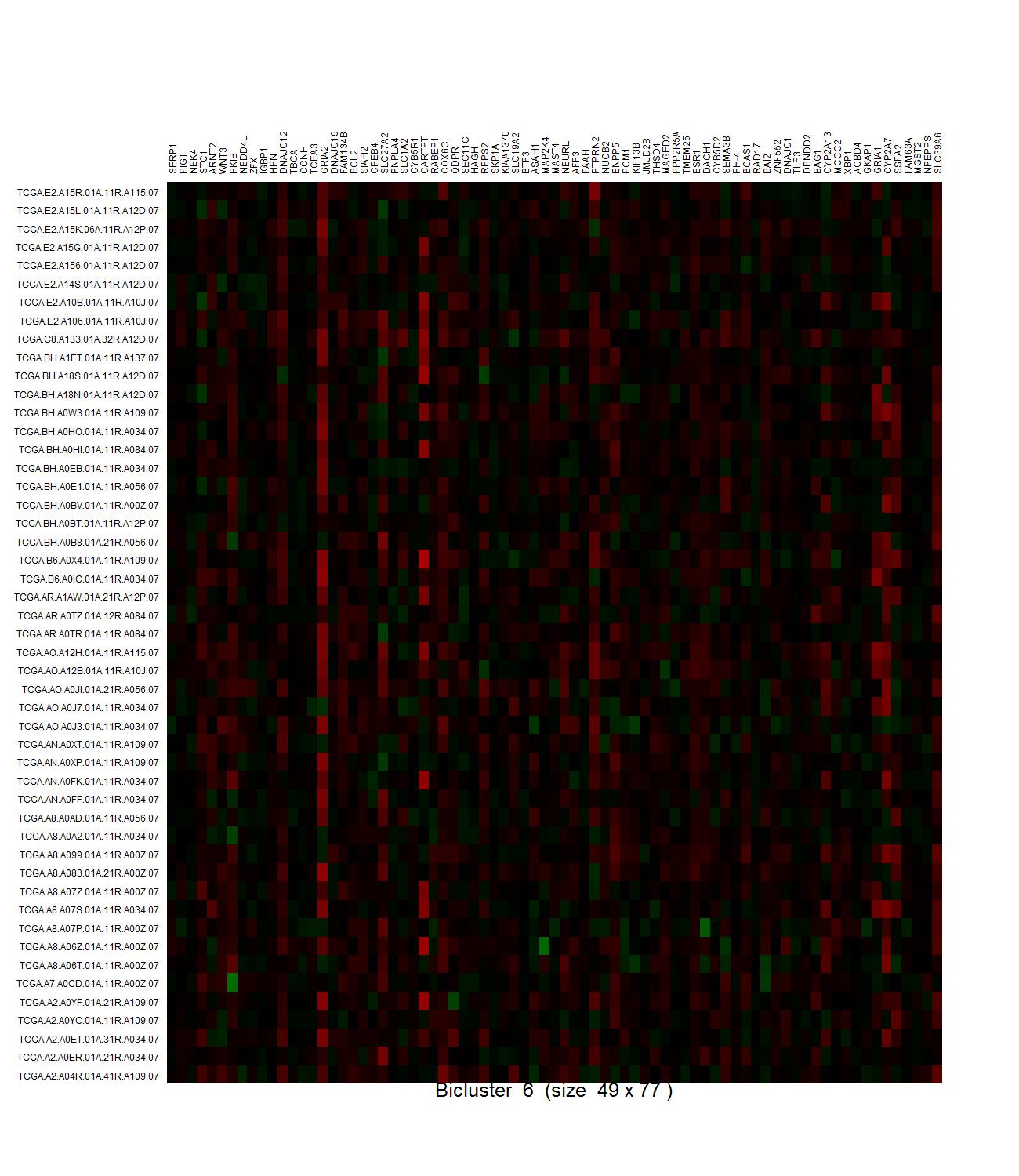

Supplement: Supplementary file 1 — The compressed file includes nine heatmap figures for the nine biclusters obtained by AP-ISA. (ZIP 1803 kb) [file 12859_2017_1926_MOESM1_ESM.zip › heatmap Figure 1-9/Figure S6.jpg]

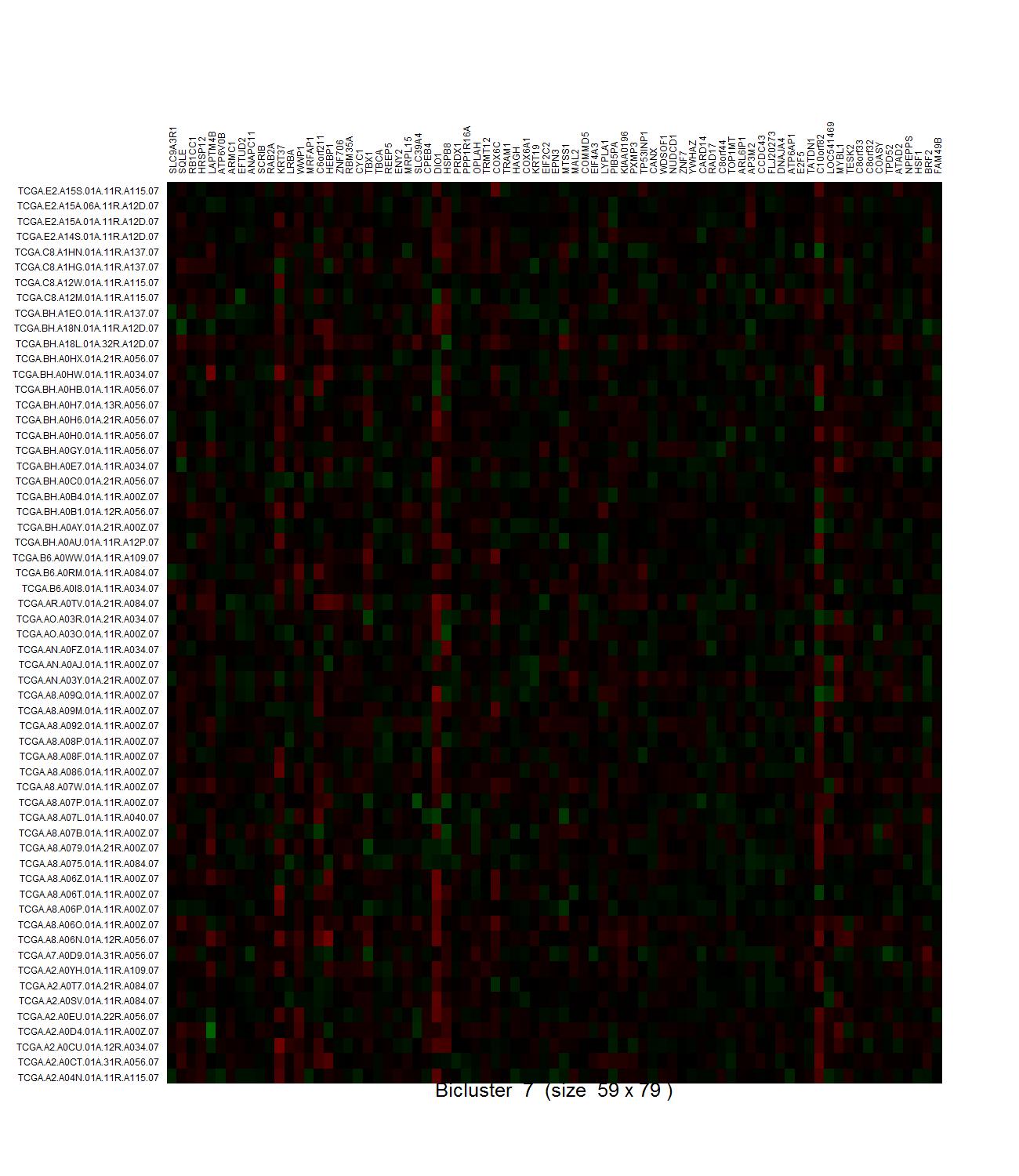

Supplement: Supplementary file 1 — The compressed file includes nine heatmap figures for the nine biclusters obtained by AP-ISA. (ZIP 1803 kb) [file 12859_2017_1926_MOESM1_ESM.zip › heatmap Figure 1-9/Figure S7.jpg]

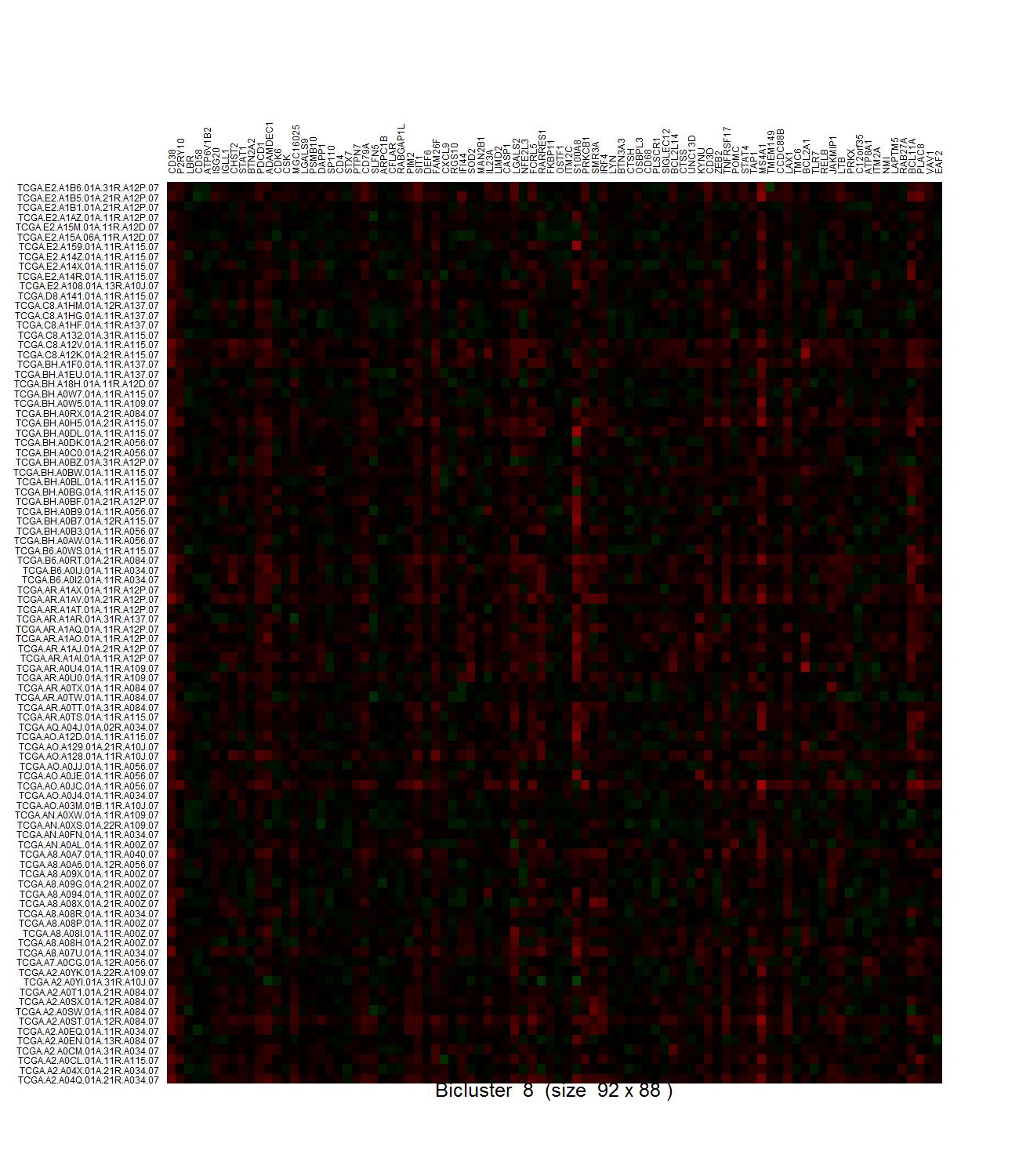

Supplement: Supplementary file 1 — The compressed file includes nine heatmap figures for the nine biclusters obtained by AP-ISA. (ZIP 1803 kb) [file 12859_2017_1926_MOESM1_ESM.zip › heatmap Figure 1-9/Figure S8.jpg]

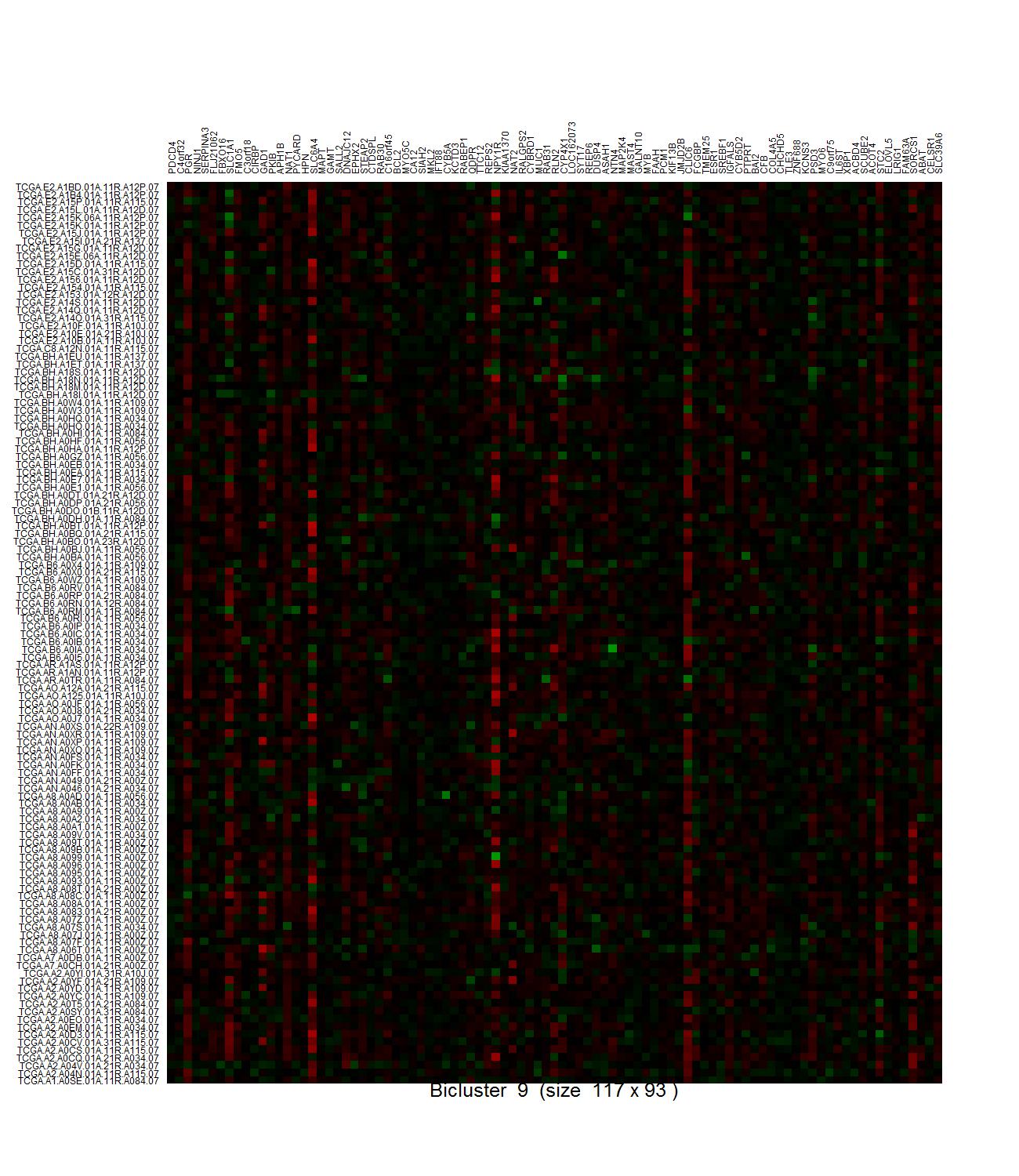

Supplement: Supplementary file 1 — The compressed file includes nine heatmap figures for the nine biclusters obtained by AP-ISA. (ZIP 1803 kb) [file 12859_2017_1926_MOESM1_ESM.zip › heatmap Figure 1-9/Figure S9.jpg]
